# Nonlinear associations between working hours and overwork-related cerebrovascular and cardiovascular diseases (CCVD)

**DOI:** 10.1038/s41598-018-28141-2

**Published:** 2018-06-26

**Authors:** Ro-Ting Lin, Lung-Chang Chien, Ichiro Kawachi

**Affiliations:** 1 Department of Occupational Safety and Health, College of Public Health, China Medical University, Room 1610, No. 91 Hsueh-Shih Road, Taichung, 40402 Taiwan; 2 Epidemiology and Biostatistics, Department of Environmental and Occupational Health, University of Nevada, Las Vegas, School of Community Health Sciences, 4505 S. Maryland Parkway, Las Vegas, NV 89154 United States; 3 Department of Social and Behavioral Sciences, Harvard T.H. Chan School of Public Health, Boston, 677 Huntington Avenue, Kresge Building, 7th Floor, Boston, MA 02115 United States

## Abstract

Long working hours are recognized as a risk factor for cerebrovascular and cardiovascular diseases (CCVD). We investigated the relationship between working hours and different CCVD severity outcomes—death, disability, and illness—across industries in Taiwan from 2006 to 2016. We applied a generalized additive mixed model to estimate the association between working hours and the rate of each severity outcome, adjusted for salary, unemployment rate, time, and a random intercept. Industry-average working hours were significantly associated with each outcome level of overwork-related CCVD, especially when monthly working hours increased from 169 (relative risk [RR] = 1.46, 95% confidence interval [CI] 1.002–2.12) to 187 (RR = 5.73, 95% CI 3.61–9.08). Although RR trends declined after monthly working hours exceeded 187, excess risks remained statistically significant. Each 1-hour increase in working hours had a stronger effect on the RR increase in death and disability than on illness. Variations in CCVD risks existed across industries, with the highest risk in transportation and information. Reducing working hours is essential to preventing overwork-related CCVD, especially the more severe outcomes. We recommend further research to address possible underreporting of less severe cases, and to explore actions to narrow the gaps in risk across industries.

## Introduction

Overwork has been recognized as a cause of sudden death from cerebrovascular and cardiovascular diseases (CCVD) as well as a cause of permanent disability or sickness caused by CCVD^1,2^. Hence, CCVD is now recognized as an occupational disease.

The most widely adopted quantitative measure used to define whether CCVD are overwork-related is the number of working hours per month^3,4^. In 1960, Buell and Breslow reported that long working hours increased the risk of mortality from coronary health diseases among males in the United States^5^. In the 1970s, sudden deaths related to overtime work began to be recognized in Japan, where the word *karoshi* was introduced to describe sudden death from overwork^6^. Since then, overwork problems have increased in other Asian countries with booming economies^7–9^. In 2015, Kivimäki and colleagues further reported a graded relation between working hours and stroke risk in Europe, the United States, and Australia^3^.

However, many existing studies have been hampered by limited data, such as self-reported rather than actual working hours and lack of expert case-by-case reviews to exclude personal and other workplace risk factors^3,10^. For example, long working hours have been associated with exposure to smoking, coffee, alcohol, unhealthy diet, and lack of exercise^11–14^. These lifestyle factors may affect risk factors for CCVD, such as blood pressure and cholesterol^11–14^. To elucidate the relationship between working hours and CCVD, it is thus important to carefully consider individuals’ personal characteristics (e.g., age, sex, family history, lifestyle risk factors) as well as their workplace risk factors (e.g., noise, chemical exposures)^4^. In particular, to ensure valid recognition of CCVD as an occupational disease—and as related to overwork—the process should involve occupational physicians and industrial hygienists.

Establishing recognition criteria for overwork-related CCVD can help decision makers avoid bias, filter out non-hours-related factors, and ensure that CCVD cases where working hours do play a role are recognized and reported. Currently, only 3 countries in the world have national criteria for recognizing and compensating overwork-related CCVD that include clear cut-off points for overtime hours worked^15^. The Ministry of Health, Labour and Welfare of Japan, in 2001, was the first to add overtime hours worked to their existing criteria as a risk factor for work-related CCVD. Similar criteria were established in Taiwan in 2004 and in South Korea in 2013^1,4,15,16^. According to these criteria, *overtime* refers to the hours worked in excess of the standard maximum monthly working hours^4^. For example, in Japan (amendment of 2001) and Taiwan (amendment of 2010), the onset of a CCVD can be attributed to overwork, after excluding personal and other workplace risk factors, if an employee (1) worked 100 hours or more of overtime during the month prior to the event, or (2) worked an average of 45 hours or more of overtime per month for 2 to 6 consecutive months prior to the event^1,15^. For industries where working overtime remains common, the risk of overwork-related CCVD is expected to be higher than in other industries. We previously argued that the introduction of specific hours-related criteria in Japan and Taiwan could help increase these countries’ ability to recognize overwork-related CCVD cases^17^.

Our preliminary investigations indicated positive associations between overwork-related CCVD and working hours^17^. However, the dose-response relationship between working hours and different types of overwork-related CCVD remained unexplored. Therefore, this study aimed to investigate the association between working hours and different severity levels of overwork-related CCVD (i.e., death, disability, and illness) by industry sector. We hypothesized that the risk of overwork-related CCVD would not differ across industries when total working hours were considered. The research goal was to provide more robust evidence of the risk of overwork-related CCVD across industries. We expect the results of this study to raise concerns about overwork culture, especially among industries whose employees often work overtime and are thus vulnerable to overwork-related diseases such as CCVD.

## Methods

We performed an ecological study that used compensation data from the Bureau of Labor Insurance, Ministry of Labor of Taiwan^18^. The Ministry of Taiwan publishes aggregate data, at the industry level, on the number of overwork-related CCVD at each severity level. Thus, the industry sector was the smallest unit of data available for analysis. The dataset contains 3 severity outcomes for overwork-related CCVD (i.e., death, disability, and illness), along with year of recognition, and the total number of person-cases of overwork-related CCVD in each industry sector. The data were linked to indicators of working conditions in each sector.

Taiwan’s Standard Industrial Classification System, Revision 10, identifies 19 industry sectors. We excluded 3 sectors (agriculture, forestry, fishing and animal husbandry; education; and public administration and defense/compulsory social security) from the study due to lack of complete data. In addition, because of changes in the classification system over time, some of the 16 sectors included in the study had merged data but no individual data available. We thus grouped the 16 sectors into 13 industry groups (Table 1). These 13 groups accounted for 88% of the employees of all industry sectors in Taiwan in 2016^19^.

**Table 1:** Grouping of industry sectors, with number of employees per group.

Group	Industry sector	Average # of employees per year, 2006–2016
Mining	Mining and quarrying	4,455
Manufacturing	Manufacturing	2,658,091
Public supply	Electricity and gas supply	93,818
	Water supply and remediation activities	
Construction	Construction	711,455
Trade	Wholesale and retail trade	1,062,182
Accommodation and food	Accommodation and food service activities	436,182
Transportation and information	Transportation and storage	525,000
	Information and communication	
Finance and insurance	Financial and insurance activities	414,182
Real estate	Real estate activities	75,455
Professional	Professional, scientific, and technical activities	264,091
Health and welfare	Human health and social work activities	363,091
Art	Arts, entertainment, and recreation	84,091
Other	Support service activities	551,364
	Other service activities	

The health outcome of interest was overwork-related CCVD. Prior to 31 December 2004, the Ministry of Labor’s criteria included 10 types of CCVD that could be recognized as related to overwork: sudden cardiac death, acute myocardial infarction, acute heart failure, dissecting aneurysm of the aorta, cerebral hemorrhage, cerebral thrombosis, cerebral embolism, subarachnoid hemorrhage, cerebral infarction, and brain damage caused by severe hypertension. After 17 December 2010, 11 types of CCVD could be recognized: myocardial infarction, acute heart failure, dissecting aneurysm of the aorta, angina pectoris, cardiac arrest, sudden cardiac death, serious cardiac arrhythmia, cerebral hemorrhage, cerebral infarction, subarachnoid hemorrhage, and brain damage caused by severe hypertension^1,17^.

We obtained data, from reports to the labor insurance system, on the annual number of person-cases of overwork-related CCVD in 3 different severity outcomes (death, disability, and illness) for each industry group. Across all industry sectors in Taiwan from 2006 through 2016, there were 619 person-cases of overwork-related CCVD, out of 91 million employee-years^18,19^. After excluding the 3 industry sectors without complete data, the cumulative number of overwork-related CCVD cases among the 13 industry groups in this study was 594 person-cases.

We also collected the number of hired employees in each industry group from the Ministry of Labor of Taiwan^19^. Hired employees are defined as those employed for salary or other economic compensation, and thus are exposed to working hours and at risk of overwork-related CCVD. We calculated the crude rate of overwork-related CCVD for each industry group by treating the total number of overwork-related CCVD in each year as the numerator and the total number of hired employees in the corresponding year as the denominator.

Overwork-related CCVD accounted for 10% of total cases of occupational disease (including death, disability, and illness) in Taiwan in 2016, but for up to 81% of deaths due to occupational diseases. The risk of CCVD among hired employees is thus worth investigating, despite the small number of cases overall.

In the process of defining a CCVD as overwork-related for the labor insurance system, occupational physicians are expected to evaluate and exclude the potential contribution to risk of employees’ personal characteristics, including age, sex, and health behaviors (e.g., drinking alcohol, smoking, and using drugs)^1^. Only exposure to the listed occupational factors, such as overtime working hours exceeding a certain level and psychological stress exceeding a certain level, are recognized in the data as risk factors for developing overwork-related CCVD^1^. Working hours is the directly measurable indicator of overtime. However, we included 2 ecological variables of working conditions—industry-specific salary and industry-specific unemployment rate—as proxy indicators of the relative state of psychological factors, as described in a previous paper^17^.

Data representing average working conditions for each industry sector were obtained from the Ministry of Labor of Taiwan^19^. The primary exposure of interest was working hours. Data included (1) regular working hours, defined as the usual hours that employers expected their employees to work per month; (2) overtime hours, defined as working hours in excess of the regular working hours per month; and (3) total working hours (per month), defined as the sum of 1 and 2. We also included in the analysis several potential confounders that had been identified in our previous study^17^, including the date of implementation of recognition criteria for overwork-related CCVD in Taiwan (2010), average industry-specific salary per month (measured in 1,000 New Taiwan Dollars), and average industry-specific unemployment rate (measured as the number of people actively looking for a job as a percentage of the labor force).

We applied a generalized additive mixed model to estimate the association between working hours and the rate of each severity level of overwork-related CCVD separately. Suppose Y_it_ is the number of cases of overwork-related CCVD at calendar year t in country i, then Y_it_ follows a Poisson model with a mean parameter λ_it_. The model is formulated as

$$\mathrm{log}({{\rm{\lambda }}}_{{\rm{it}}})={\rm{intercept}}+{\boldsymbol{\beta }}\times {\bf{X}}+{\rm{f}}({{\rm{Hour}}}_{{\rm{it}}})+{{\rm{\gamma }}}_{{\rm{i}}}+{\rm{offset}},\,{\rm{t}}=1,\,\ldots ,\,11;i=1,\,\ldots ,\,13,$$

where **β** is a coefficient vector for the linear independent variables in the vector **X**, including calendar year, the status of implementation of the new criteria or note, salary, and unemployment rate. A penalized cubic spline f(Hour_it_) was adopted to examine the nonlinear association between working hours and overwork-related CCVD. A random intercept γ_i_ at the level of industry group was appended in the model to take the heterogeneity among industries into account. The last offset term is the logarithm of employees in each year and each country.

The estimated cubic spline can be transformed into relative risk (RR) to demonstrate the excessive risk of overwork-related CCVD related to a specific number of working hours compared to the risk at the smallest number of working hours among the studied industries. Similarly, the random intercept at the industry group level can be transformed into RR to examine the excessive risk of overwork-related CCVD in each industry compared to the average of all the included industries. We performed the statistical analyses using RStudio version 1.0.143 (RStudio, Boston, Massachusetts, United States). The significance level is 0.05.

### Ethical approval

This study has been by the Research Ethics Committee, China Medical University & Hospital, Taichung, Taiwan (No. CMUH106-REC1-010).

### Data availability

The datasets generated during and/or analyzed during the current study are publicly available from the website linkages in references.

## Results

Among the 13 industry groups studied for 2006–2016, the manufacturing group accounted for the largest proportion of the cumulative number of overwork-related CCVD cases (27.6%; Table 2). The next highest proportions were found in the “other” group (22.4%) and the transportation and information group (16.8%). The industries’ average number of annual CCVD cases shows a similar pattern. In terms of the rate of overwork-related CCVD, the mining group had the highest rate (30.3 per million employees per year), but it had only 2 cases. The second highest rate was found in the “other” group (21.1 per million employees per year), followed by the transportation and information group (16.9 per million employees per year), and the real estate group (14.9 per million employees per year). The 2 industry groups with the lowest rates were the finance and insurance group (1.1 per million employees per year) and the accommodation and food group (2.3 per million employees per year).

**Table 2:** Working hours and rates of overwork-related CCVD by industry group, 2006–2016.

Industry group	Overwork-related CCVD				Working hours
	Cumulative number of cases	Proportion of cases in all industries	Average cases per year (SD)	Average rate per year per million employees (SD)	Average working hours per month (SD)
Mining	2	0.3%	0.2 (0.4)	30.3 (67.4)	181.4 (3.3)
Manufacturing	164	27.6%	14.9 (9.0)	5.5 (3.3)	183.8 (4.0)
Public supply	5	0.8%	0.5 (0.7)	4.5 (6.9)	176.7 (2.5)
Construction	40	6.7%	3.6 (3.2)	5.0 (4.4)	175.4 (3.5)
Trade	86	14.5%	7.8 (3.3)	7.3 (3.0)	173.2 (3.4)
Accommodation and food	11	1.9%	1.0 (1.3)	2.3 (3.1)	170.0 (2.8)
Transportation and information	100	16.8%	9.1 (5.5)	16.9 (10.0)	173.2 (2.4)
Finance and insurance	5	0.8%	0.5 (0.9)	1.1 (2.2)	166.3 (2.2)
Real estate	13	2.2%	1.2 (1.3)	14.9 (16.8)	179.0 (3.8)
Professional	12	2.0%	1.1 (1.0)	4.2 (4.3)	173.1 (3.0)
Health and welfare	21	3.5%	1.9 (1.6)	5.0 (3.8)	173.3 (2.6)
Art	2	0.3%	0.2 (0.6)	2.5 (8.3)	180.9 (4.5)
Other	133	22.4%	12.1 (9.6)	21.1 (15.9)	192.2 (4.1)

CCVD = cerebrovascular and cardiovascular diseases. SD = standard deviation.

The longest average working hours per month were found in the “other” group (192.2 hours; Table 2), followed by the manufacturing group (183.8 hours) and the mining group (181.4 hours). The 2 industry groups with the lowest rate of overwork-related CCVD also had the lowest average working hours per month—166.3 in the finance and insurance group and 170.0 in the accommodation and food group.

Table 3 shows the linear association between each CCVD severity outcome and each covariate. The implementation of detailed recognition criteria for overwork-related CCVD in 2010 was a significant predictor of the rates of total overwork-related CCVD and of death and illness due to overwork-related CCVD. A parameter estimate of 0.976 suggests that after the 2010 criteria were implemented, the rate of total overwork-related CCVD increased 2.7-fold (${e}^{0.976}$, 95% confidence interval [CI] 1.7–4.0). Similarly, after 2010, the rates of death and illness from reported overwork-related CCVD increased 4.2-fold (${e}^{1.427}$, 95% CI 2.3–7.6) and 2.2-fold (${e}^{0.780}$, 95% CI 1.2–4.0), respectively. No significant association was observed between the severity outcomes and salary, unemployment rate, or time.

**Table 3:** Parameter estimates of covariates of each severity outcome of overwork-related CCVD by outcome, adjusted for working hours and a random intercept.

Outcome of overwork-related CCVD	Criteria (after vs. before 2010 criteria)			Average monthly salary (per 1,000 New Taiwan Dollars)			Unemployment rate (%)			Time (calendar year since the 2006 baseline year)		
	Estimate	SE	P-value	Estimate	SE	P-value	Estimate	SE	P-value	Estimate	SE	P-value
Total	0.976	0.213	<0.001	0.011	0.018	0.535	0.075	0.076	0.329	0.018	0.039	0.653
Death	1.427	0.308	<0.001	−0.002	0.023	0.936	0.165	0.104	0.116	−0.063	0.057	0.271
Disability	0.521	0.394	0.188	0.008	0.022	0.717	−0.133	0.156	0.398	0.097	0.069	0.159
Illness	0.780	0.305	0.012	0.019	0.024	0.439	0.118	0.105	0.260	0.058	0.056	0.297

CCVD = cerebrovascular and cardiovascular diseases. SE = standard error.

**Figure 1:**
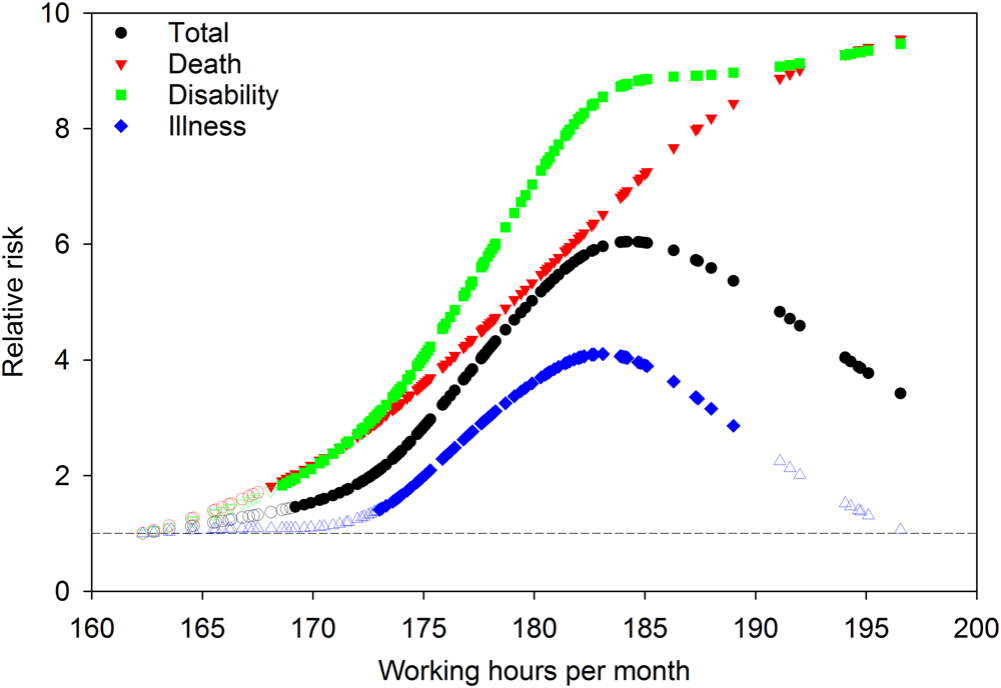
Smoothing functions for the relationship between working hours and overwork-related CCVD by severity outcome, adjusted for criteria implementation, average monthly salary, unemployment rate, time, and a random intercept. The dashed line indicates relative risk (RR) equal to 1. Shaded symbols indicate RRs that are significantly greater than 1; unshaded symbols indicate RRs that are not significantly greater than 1.

Number of working hours was found to be a significant nonlinear predictor of each severity outcome of overwork-related CCVD, with different relationship curves for each outcome (Fig. 1). For total overwork-related CCVD, the RR increased sharply from 1.46 (95% CI 1.002–2.12) to 5.73 (95% CI 3.61–9.08) when working hours increased from 169.2 to 187.3 hours per month. While the RR declined again for monthly hours exceeding 187.3 hours, it remained both greater than 1 and significant.

For death from overwork-related CCVD, the RR had a monotonic increase, and was significantly greater than 1 only after the monthly working hours exceeded 168.1. The risk of death reached its maximum when monthly working hours reached 196.55 (RR = 9.55; 95% CI 2.87–31.75). For disability from overwork-related CCVD, the RR significantly increased from 1.83 (95% CI 1.03–3.25) at 168.6 hours per month to 8.72 (95% CI 5.20–14.63) at 183.9 hours per month, at which point it reached a plateau with only incremental increases at 184 working hours or more. For illness from overwork-related CCVD, the RR was significantly greater than 1 only when monthly working hours ranged between 173 and 191.1. The highest risk of illness was observed at 183.1 working hours per month (RR = 4.10; 95% CI 2.53–6.64; Fig. 1).

**Figure 2:**
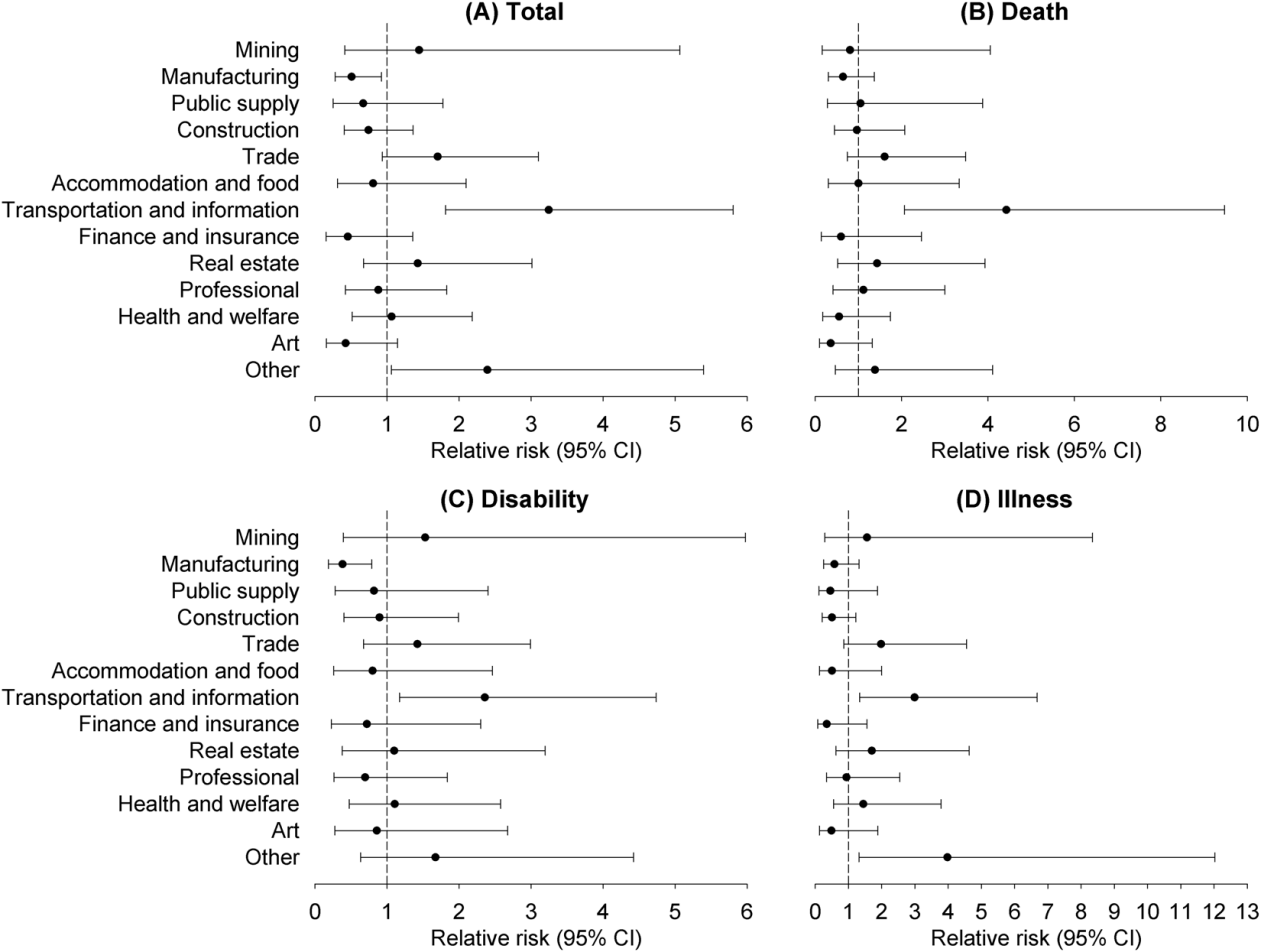
Relative risks of overwork-related CCVD by severity outcome and industry group, compared to the average rate of CCVD, adjusted for working hours, criteria implementation, average monthly salary, unemployment rate, and time.

We also compared the RR of total overwork-related CCVD for respective industry groups to the average across all industry groups. The highest RR was observed in the transportation and information group (3.2, 95% CI 1.8–5.8; Fig. 2A), followed by the “other” group (2.4, 95% CI 1.1–5.4). The lowest RR was observed in the manufacturing group (0.5, 95% CI 0.3–0.9). The transportation and information group was the only group with RRs higher than the average in each severity outcome—its RRs ranged from 2.4 (95% CI 1.2–4.7) to 4.4 (95% CI 2.1–9.5) across CCVD outcomes (Fig. 2B–D). The “other” group had a RR significantly higher than the average (4.0, 95% CI 1.3–12.0) in illness from overwork-related CCVD (Fig. 2D). The manufacturing group had a RR significantly lower than the average (0.4, 95% CI 0.2–0.8) in disability from overwork-related CCVD (Fig. 2C).

## Discussion

In this ecological study, we found a nonlinear relationship between working hours and total overwork-related CCVD, with a significantly higher risk of CCVD (RRs) found in industry groups where employees worked 169.2 hours or more per month compared to industry groups where employees worked 162.3 hours per month. Furthermore, the degree of risk differed for each CCVD severity outcome, with a greater likelihood of overwork-related CCVD resulting in death or disability versus illness when monthly working hours increased.

This nonlinear relationship could be explained in 2 ways. First, total working hours is a potential predictor of the severity of overwork-related CCVD. As working hours exceed 183 hours per month, the risk of death from CCVD increases, whereas the risk of disability remains flat and the risk of illness decreases. Second, more severe cases of CCVD are more likely to be reported and then recognized as related to overwork^20^.

Our findings regarding a graded increase in both death and disability from overwork-related CCVD among industry groups are consistent with results reported by previous meta-analyses and studies at the individual level^3,21,22^. Less severe CCVD cases (e.g., illness) are more likely to be recorded and covered under Taiwan’s national health insurance system than its labor insurance system^23^, which was the source of our data. Wang *et al*. compared the inpatient and outpatient expenses of occupational injuries and diseases before and after 1995, the year that Taiwan introduced national health insurance system^23^. The inpatient expenses of occupational injuries and diseases didn’t change a lot, whereas the outpatient expenses reduced rapidly—the expenses after 2000 was only one-third of the expenses before 1995^23^. Application procedures for occupational diseases in the labor insurance system are more tedious, and workers know that the national health care system can cover some of their medical expenses. Workers or families who are not pursuing additional compensation may be less motivated to apply to the labor insurance system, and particularly for less severe cases^24^. By differentiating overwork-related CCVD cases according to severity, this study was able to detect these possible causes of underreporting.

Equity regarding working hours and regulations for maximum working hours are widely debated^25^, particularly for employees in industries that rely on shift work or prolonged working hours^26^. However, our study found that even after adjusting for working hours and other covariates, the risk of overwork-related CCVD at each level of severity was consistently higher in the transportation and information sectors. This variation may be driven by greater public attention to issues of overtime in these sectors. For example, overwork among professional drivers has had a direct impact on public road safety^27^, with news media covering drivers who suffer from sudden onset of CCVD and cause traffic accidents. Moreover, due to the increasing number of these cases among professional drivers in Taiwan, the government has targeted the transportation and information industry sectors for labor inspection in recent years. Greater public attention could thus lead to greater recognition of CCVD cases as related to overwork and less likelihood of such cases being underreported.

Two potential limitations of this study should be addressed. First, public data with respect to CCVD and working hours were available only at the industry sector level. To avoid the possibility of ecological fallacy^28^, we avoided making inferences at the individual level. However, because we obtained our data from a single authoritative database, comparability of data across industry sectors was enhanced, and consistency of indicators was assured. Our findings at the industry sector level did not contradict the conclusions in the literature reported at the individual level^3,10^.

Second, the minimum and maximum working hours available in our data were 162.3 hours per month (or 40.6 hours per week) and 196.6 hours per month (or 49.2 hours per week), respectively. We could not extrapolate our results to industry sectors in which employees worked less or longer than this range of hours. Similarly, our findings of dose-response relationship cannot be extrapolated to industry sectors outside of the 16 industry sectors included in the analysis (Table 1). For example, burnout has become a widespread concern among public servants^29^. Including additional industry sectors, particularly those with long working hours, in future research could provide more comprehensive coverage of the dose-response relationship and better understanding of gaps in risks across industries.

Despite the above-mentioned limitations, our study has several strengths. First, the CCVD included in this study are overwork-related cases that were officially recognized under the occupational disease compensation scheme of the labor insurance system, not self-reported cases or CCVD diagnoses drawn from a general health dataset. Thus, non-occupation-related factors had already been excluded for each case by occupational physicians. Second, by applying a cubic spline and differentiating the severity outcomes, we were able to observe different nonlinear associations between working hours and each outcome level of overwork-related CCVD. Our findings are thus able to specify a nonlinear relationship. To reduce the burden of overwork-related CCVD in Taiwan, national policy must shorten working hours and narrow the gaps in risks among industries.

## Conclusion

In conclusion, number of working hours has a significant nonlinear association with overwork-related CCVD. However, the risk pattern identified is quite different for each severity level of CCVD, from illness to disability to death. Enforcing an upper limit to monthly working hours may help decrease the risk of CCVD-related death. We also recommend further research to address possible underreporting of less severe cases of CCVD, as well as to explore what actions should be taken to narrow the gaps in risk across industries.
